# 3D biofabrication for tubular tissue engineering

**DOI:** 10.1007/s42242-018-0013-2

**Published:** 2018-05-23

**Authors:** Ian Holland, Jack Logan, Jiezhong Shi, Christopher McCormick, Dongsheng Liu, Wenmiao Shu

**Affiliations:** 1 Department of Biomedical Engineering, University of Strathclyde, Glasgow, G1 1QE UK; 2 Department of Chemistry, Tsinghua University, Haidian, Beijing 100084 People’s Republic of China

**Keywords:** Tubular organs, Tissue engineering, 3D printing, Bio-inks

## Abstract

The therapeutic replacement of diseased tubular tissue is hindered by the availability and suitability of current donor, autologous and synthetically derived protheses. Artificially created, tissue engineered, constructs have the potential to alleviate these concerns with reduced autoimmune response, high anatomical accuracy, long-term patency and growth potential. The advent of 3D bioprinting technology has further supplemented the technological toolbox, opening up new biofabrication research opportunities and expanding the therapeutic potential of the field. In this review, we highlight the challenges facing those seeking to create artificial tubular tissue with its associated complex macro- and microscopic architecture. Current biofabrication approaches, including 3D printing techniques, are reviewed and future directions suggested.

## Introduction

Tubular tissue structures are ubiquitous throughout the body and its organ systems, with notable examples found in the vasculature (arteries, veins, capillaries), respiratory, (oesophagus, trachea), urinary (ureter, urethra, bladder), and gastrointestinal systems [1]. Whilst shape is the common feature amongst these cited examples, there is considerable variation in scale, tissue architecture and function that are ultimately imbued by the arrangement of different cell types and their surrounding extra cellular matrix (ECM). In a similar fashion to other organ systems, tubular tissue is prone to disease and malfunction, often requiring therapeutic intervention in the form of replacement with a synthetic prothesis, donor tissue or an autologous implant [2, 3]. However, these procedures currently have a number of limitations. There remains a disparity between the high demand for replacement donor tissue and the paucity of suitable donors [4, 5] and adequate autologous tissue may not be always be available [6–9]. Synthetic polymer, prostheses, whilst readily available, generally struggle to match demanding anatomical and mechanical requirements, have limited growth potential and have persistent concerns around their long-term patency [6, 8, 10].

Tissue engineering has emerged as one of the healthcare technologies of the future and offers a potential route to address the current therapeutic challenges by allowing researchers and clinicians to create patient-specific devices that more accurately represent the in-vivo tissue being replaced or augmented [9, 11]. The advent of bioadditive manufacturing in recent years has further added to the “technological toolbox” that is available to those engaged in tubular tissue engineering, thus opening up a plethora of clinically driven research opportunities [12]. Specifically, it has enabled researchers and clinicians alike to consider the possibility of creating prostheses that closely mimic the native architecture of the patient’s anatomy at both a macro- and microscopic level [13–15]. It is therefore envisaged that such artificial devices, created in the laboratory, can be bespoke to the therapeutic and anatomical requirements of the patient, thus improving clinical outcomes. In this regard, tissue engineering, achieved through additive biomanufacturing, can be considered part of the wider healthcare trend towards personalised medicine. In addition to their use as prostheses, accurate anatomical tubular organ reconstructions have also recently been proposed as a method for improved consultations between surgeons and their patients, in clinical training scenarios and planning complex surgical operations [16].

Research using 3D biofabrication methods to create vasculature predominates over other tubular tissue types. Vasculature replacement or augmentation is utilised to treat a range of conditions, including aortic aneurysm repair and congenital defects. Vascular networks are also critical for researchers seeking to create any large artificial organ system, allowing the nutrient and gas exchange processes that are necessary in an in-vivo environment for tissue survival [17, 18]. Within the domain of vascular tissue engineering, the challenge resides in creating the distinct endothelial, medial and adventitial layers that are themselves composed of smaller concentric rings of cells and ECM. The task is further complicated by the high level of heterogeneity across the vascular system, not only in terms of the structural arrangement and ratios of these layers but also in the phenotype of the constituent cells (Aird 2007). The target for research groups is to create anatomically accurate, branched, vasculature that has the representative thin collagen and elastin layers, containing the requisite cells in sufficient numbers and alignment [10, 19].

There is also need for the development of tubular structures in other areas of healthcare. Examples include oesophagus and trachea repair following disease, where the airways require reconstruction. Artificial protheses created in the laboratory could enable the augmentation or replacement of ineffectual or collapsed airways, replacing current stenting techniques. The capability to match the device to the complex anatomical geometry seen in the tracheobronchial tree is a distinct advantage for bioadditive manufacturing techniques over the use of standardised prostheses [20, 21]. Whilst individual studies are often focussed on the creation of a specific tubular tissue type, the methodologies that they employ could be applied to other organs requiring transplantation. Therefore, whilst many of the methods described in this review refer to studies aiming to recreate vascular tissue, the principles and techniques used can be applicable to the creation of other tubular tissue structures found throughout the body.

In this article, we aim to review current 3D biofabrication methodologies and techniques used for tubular organ construction and provide an overview of 3D printing technologies and materials that are of relevance in the development of tubular structures.

## Current tissue engineering approaches

**Fig. 1:**
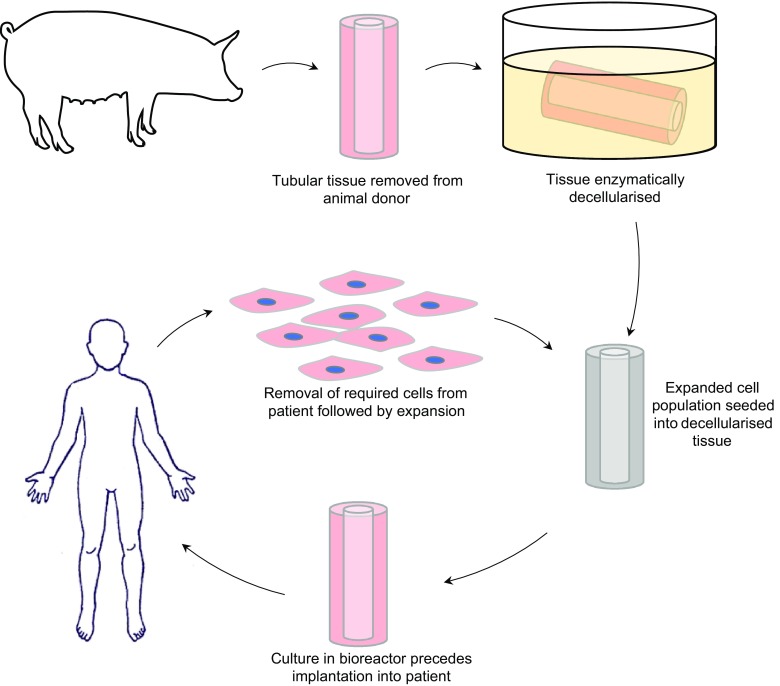
Tissue engineering process for tubular organs using decellularised animal donor tissue

One method that has attracted widespread attention for tissue engineering tubular organs is the use of a scaffold material that can then be subsequently populated with the patient’s own cells. The intention is to create a device that has the requisite 3D ECM and patient-specific cell types needed to create functional tissue that has a reduced risk of autoimmune rejection (Fig. 1). The use of this process has been explored for vascular tubes using a range of sources for the scaffold such as synthetic polymers [22, 23], natural polymers [24, 25], decellularised animal [26] and human tissue [27]. For an extensive review on scaffold seeding, materials and methods for vascular tissue engineering refer to Pashneh-Tala et al. [2]. The decellularisation and recellularisation approach has also been attempted for bile duct replacement in a murine model [28]. Tracheal replacement too has utilised this method, although simple tubular construction is not sufficient to accurately represent the in-vivo tissue, with it being comprised of multiple C-shaped cartilage sections [21]. However, high profile failures have limited progress in this area, with cellular infiltration and artificial organ acceptance shown to be not as successful as previously hoped [29].

**Fig. 2:**
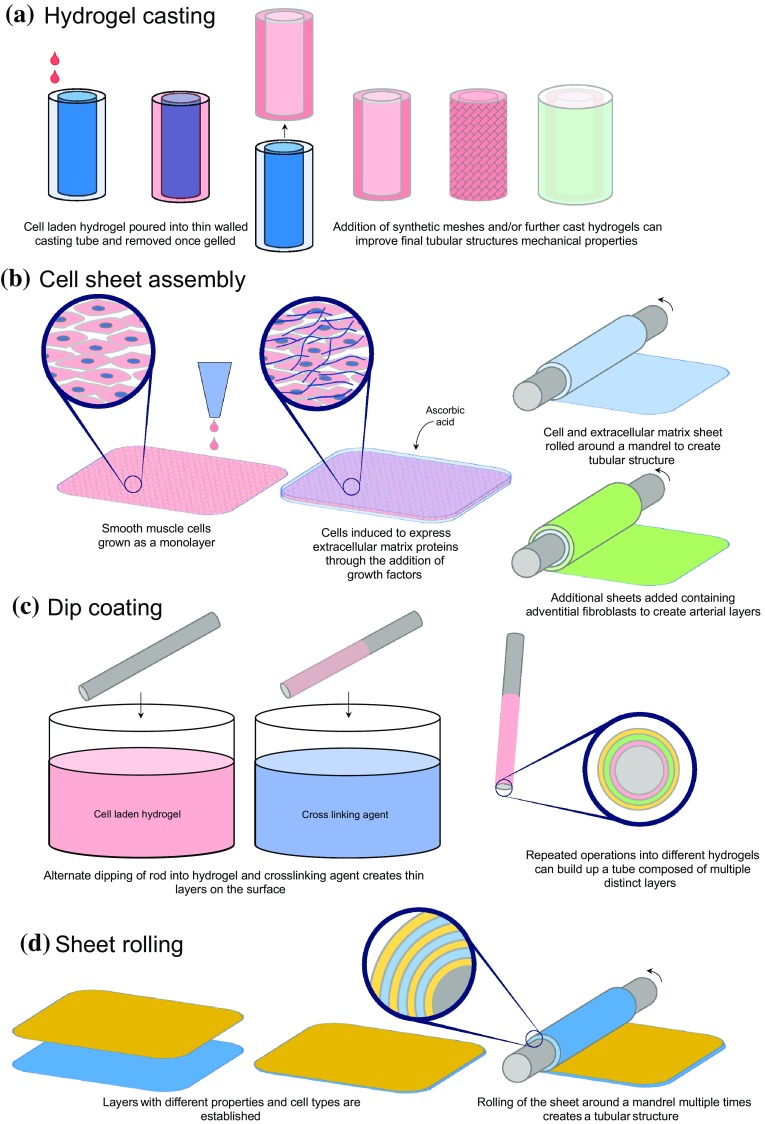
Tissue engineering variants and methods for manufacture of tubular organs. **a** Cell-laden hydrogel casting with synthetic polymer mesh [30]. **b** Cell and extracellular sheet matrix assembly [31]. **c** Rod dip coating [34, 35]. **d** Sheet rolling [32, 33]

Beyond scaffold seeding, other manufacturing techniques to create tubular organ structures have been reported and refined since the 1980s. Weinberg and Bell pioneered the tissue-engineered blood vessel in 1986, by casting collagen containing smooth muscle cells and adventitial fibroblasts into tubes (Fig. 2a). Their devices, however, required the integration of a synthetic Dacron mesh to increase the burst pressure, a deficiency the authors attributed to the ECM composition and low cell densities [30]. A further step forward in the field was developed by L’Heureux et al., who used a process that was devoid of any synthetic or exogenous biological material by inducing the excretion of collagen in cultured cell sheets of smooth muscle cells and fibroblasts (Fig. 2b). Layers of these sheets were then wrapped around a mandrel and further cultured as tubes to create a final artery mimic containing distinct endothelial, medial and adventitial layers [31]. An acellular approach to sheet assembly has been shown by Kumar et al. (Fig. 2d). The group created a collagen and elastin sheet that was then rolled as a tube to create an acellular version containing the distinct elastin and collagen layers that are observed in native arterial tissue and deemed essential for mechanical strength. Compared against traditional rigid polymer conduits, the device had comparable burst pressures and reduced adherence of platelets, a pro-thombotic mediator [32]. This rolling methodology was further developed by Othman et al., using an automated platform to create tubular architectures with desired scales and dimensions at cell resolutions. By repeating the rolling programme, three-dimensional tubular structures with multiple layers, cell types and materials could be constructed. The fabricated tissues could be directly transferred into a perfusion bioreactor without further manipulation. This strategy provides a convenient method to fabricate multiple biomaterial types and a route to high volume device production [33].

A simplified, efficient method of tubular tissue construction is the use of a rod that can be alternately dipped into a cell-laden hydrogel and a cross-linking agent (Fig. 2c). Repeated dipping operations form multiple layers that can potentially be made from different hydrogels. The method was used by Tabriz et al., to create layers containing viable human embryonic kidney and mouse fibroblast cells. The thickness of each layer was determined to be in the range 126–220 $\upmu$m with this being dictated by the wettability of the surface being coated, and the composition of the hydrogel adhered to the surface prior to the cross-linking phase. The authors conclude that their approach could be adapted for use in a range of tubular tissue types [34]. This methodology was further developed by Wilkens et al., who introduced motors to rotate and the dip rods. A major benefit of this approach was a level of control over the thickness of the layers. The smallest layer attained was $\approx$ 25 $\upmu$m and is of significance as this matches the medial collagen and smooth muscle cell layers observed in native arterial tissue [35].

Following assembly of tubular structures, a number of research groups have highlighted the influence that the post manufacture culture conditions can have on an artificially assembled construct. The most cited example is the migration and alignment of cells in vascular tissue, with created tubular organs cultured in bespoke bioreactors with integrated flow systems. It has been observed that exposing artificially engineered tissue to fluid flow induced shear stress, or mechanical stretching, can cause not only the cellular elements to migrate and align in a direction perpendicular to flow direction [7, 23, 25, 36] but also ECM components too [37]. The postassembly culture stage can therefore be exploited to remodel and mature artificial tubular organs towards a more representative in-vivo state than is feasible using the initial bioadditive assembly techniques.

Although the progress in methodology and techniques for constructing tubular organs has been made, the fabricated structures are still relatively simple in comparison with the native tissue they intend to mimic. This is especially true in regard to the control the processes have over the microscale organisation of cell and extra cellular matrix layers and in particular the complex anatomical architecture of tubular organ networks, such as multiple bifurcations, typically observed in native tubular structures. More sophisticated techniques that enable researchers to fabricate complex, multi-layered structures with precise spatial controls are therefore needed.

## 3D bioprinting

3D bioprinting [38, 39] has emerged as a one such technology that has the potential to fabricate complex structures composed of cells and an associated support matrix and can be used to create tubular organ structures. The possible modalities of 3D bioprinting can be categorised into three main categories, namely extrusion-based bioprinting (EBB), droplet-based bioprinting (DBB) and laser-assisted bioprinting (LAB) [40].

Extrusion-based bioprinting involves the positive displacement of material by an applied force, which can be either pneumatic, mechanical or solenoid-assisted. Usually non-Newtonian fluids are utilised, whereby viscosity and shear response are important; thixotropic substances are the most desirable here. It is imperative that extrudable materials can easily overcome surface tension and are capable of rapid gelation for shape retention without unwanted flow on print beds. Additionally, the substrate should have surface roughness with low wettability to allow prints to adhere to surfaces with shape retention. EBB is the most economical of the three bioprinting modalities, and there is a wide range of printable viscosities (30–$6\times 10^7$ mPa s) [41]. Printing resolution is a major technical challenge of extrusion-based bioprinting, as is the minimisation of cellular shear stress upon nozzle ejection, both of which are not mutually exclusive [42].

Secondly, droplet-based bioprinting makes use of energy sources such as electricity, acoustics and heat to create bio-ink droplets. There are four possible methodologies when applying DBB: inkjet printing (electrostatic/piezoelectric/thermal), electro-hydrodynamic jetting, acoustic droplet ejection and micro-valve printing. Bio-inks in DBB should have low viscosity and have a non-fibrous structure, to allow easy flow through tubing and nozzles, to avoid clogging. The need for low viscosity is, however, problematic as it is challenging for low-viscosity materials to change form into a solid-state structure. Bio-inks must also be rheopectic in nature to allow droplets to form upon ejection from nozzles. Further, substrates must have adequate surface tension to travel through cartridges without leakage, which can lead to print head flooding and nozzle tip wetting. Droplets should solidify upon contacting print bed surfaces to avoid unwanted material flow. There are many drawbacks to using DBB, such as low cell concentrations, thermal and mechanical stress to cells and cell encapsulation imprecision [43].

Laser-assisted bioprinting makes use of laser energy to print liquids onto supports substrates at high precision, with no need for nozzles thus avoiding the clogging issues associated with EBB and DBB. LAB can be achieved by two separate means—cell-transfer-based and photo-polymerisation LAB. Cell-transfer-based operations can involve laser-guided direct writing [44] or laser-induced forward transfer (LIFT) [45] whereby bio-ink is ejected from a reservoir to the substrate by laser, thus allowing a jet to be produced. The bio-ink in cell-transfer laser systems should be capable of adhering to substrates whilst possessing low surface tension to allow uniform spreading on the surface without dripping. In addition, the bio-ink should be capable of transforming thermal energy into kinetic energy with ease whilst also displaying high viscoelasticity and swift gelation to allow jet formation. Processes involving photo-polymerisation include stereolithography (SLA) [46] and two-photon polymerisation (2PP) [47], in which the laser beam selectively causes the solidification of a photo-curable bio-ink via polymerisation. The use of non-toxic, water-soluble photo-initiators and light absorbers allows photo-polymerisation to occur, resulting in the manufacture of tissue constructs with uniform layer thickness. The demands on bio-inks here include high mechanical strength and the ability to maintain the even distribution of cells in the precursor solution [48].

## 3D bioprinting for tubular tissue engineering

A range of approaches have used 3D printing technology to create tubular organ structures; however no methodology has emerged a forerunner. Such variation is indicative of both the nascent nature of the field and of the variety seen in the native tubular tissue that is being mimicked.

**Fig. 3:**
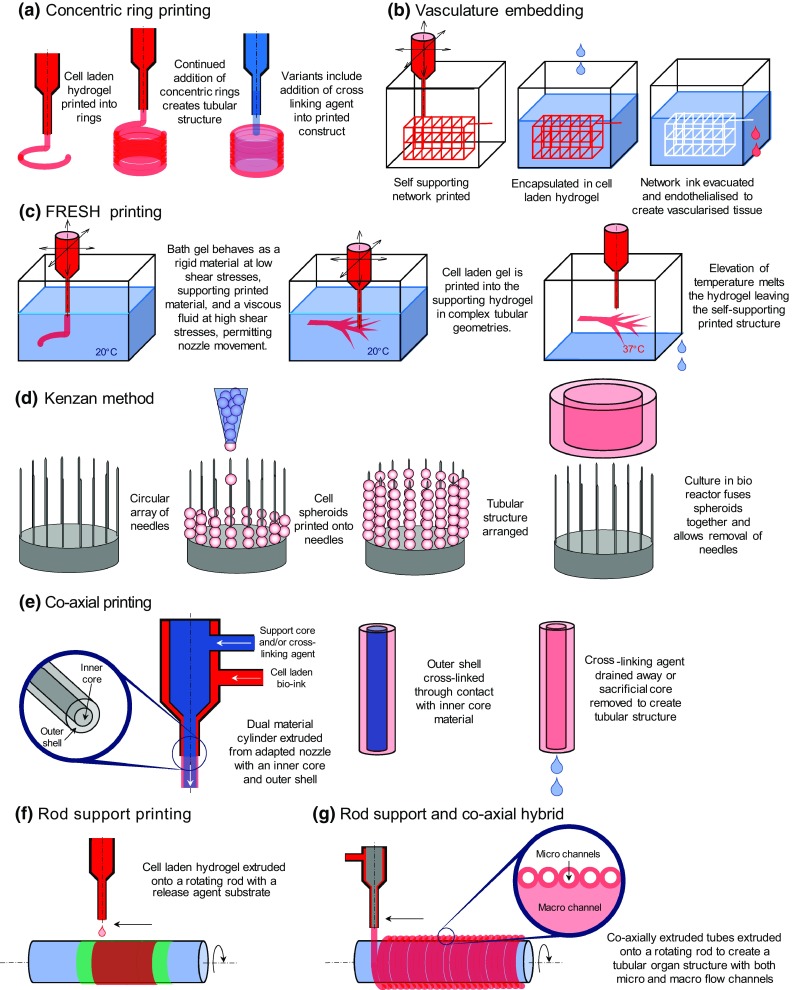
3D printing variants and methods for bioadditive manufacture of tubular organs. **a** Concentric ring assembly [49]. **b** Vasculature network creation via fugitive inks [50, 51, 53]. **c** Freeform reversible embedding of suspended hydrogels (FRESH) printing [54, 55]. **d** Kenzan printing of cell spheroids onto needles [56, 57]. **e** Coaxial tube formation from a modified nozzle [59–61]. **f** Rod support printing (Sichuan Revotek corporation, unpublished). **g** Coaxial extrusion onto rotating glass rod [62]

The use of 3D printers to arrange annuli or a continuous spiral of material that when layered in the vertical plane progressively fabricates a tubular structure (Fig. 3a). The key disadvantage is the substrate layers must be structurally rigid enough to support the both the upper layers and the addition of new material. This places mechanical constraints on the type of bio-inks that can be used and ultimately limits the dimensional range that can be created. Furthermore, the creation of multiple concentric layers composed of different hydrogel and cell types seen in native tubular tissue is difficult to achieve. Consequently, printed tubular tissues created using this method are usually composed of a single homogenous material. However, Tan and Yeong were able to induce a variation in the properties of the tube wall through the addition of a cross-linking agent to core of the tube and through careful optimisation of the viscous properties of the alginate hydrogel, print self-supporting structures with clinically relevant dimensions [49] (Fig. 3a).

Fugitive inks have been utilised to create complex tubular anatomical shapes as a means of overcoming the problems associated with viscous bio-inks that are unable to provide the structural integrity needed during the printing process. The process involves printing self-supporting structures using a fugitive ink, exploiting its rigid mechanical properties. This structure can then be embedded in a second hydrogel that contains the desired cells for the tissue type to be created. The fugitive ink is then liquified and removed through a variation in temperature or simply dissolved (Fig. 3b). The relevance of this technique to tubular organ manufacture is that the fugitive ink can be printed as a network of interlinked cylinders, which can subsequently be endothelialised to create a tubular vascular network capable of allowing nutrient perfusion to the surrounding tissue [50–52] and angiogenic sprouting [53]. In principle, this technique represents an innovative bioprinting variation on the lost wax casting method historically used to produce metallic components.

Freeform reversible embedding of suspended hydrogels (FRESH) is a novel approach that could be considered as the inverse of the fugitive ink method developed by Kolesky and others. The technique prints hydrogels directly at 20 $^{\circ }$C into a support bath containing a secondary hydrogel, also referred to as a fugitive support gel. This has the effect of providing mechanical support for the printed material. Elevating the temperature to 37 $^{\circ }$C melts the support bath hydrogel, leaving a self-supported, printed structure behind [54] (Fig. 3c). The method has been employed to print a range of complex tubular structures, including helical coils and bifurcated vessels [55].

An innovative method of tubular organ assembly has been demonstrated by Itoh et al. using metallic needles in a circular array as a temporary scaffold for supporting cell spheroids. Termed the Kenzan method, the group used the needles to support smooth muscle cell and fibroblast spheroids (650 $\upmu$m, diameter) that then, after a period of culture in a bioreactor, fused permitting removal of the support needles to create a rigid tubular structure [56, 57] (Fig. 3d). As an alternative to spheroids, Norotte et al. assembled high density extruded cellular cylinders, supported by acellular agarose rods, into tubular tissue, with this process achieving a reduction in printing times when compared to individual spheroid assembly [58].

The process of coaxial printing has also been employed by several research groups to create microtubular constructs. A nozzle is modified to include inner and outer cores that allow gels and bio-inks to be extruded into tubes, with the final size determinised by the dimensions of the nozzle. By extruding a cross-linking agent as the inner core, the outer shell can be rapidly cross linked to form a self-supporting hollow tube [59, 60]. Jia et al. also included an additional outer shell to their nozzle to allow cross-linking perfusion from two directions [61]. Gao et al further extended the principle to print two separate bio-inks of an endothelial progenitor cell-laden alginate surrounding a more rigid sacrificial supporting core, containing a cross-linking agent [62] (Fig. 3e). This method permits a longer cross-linking time, allowing a greater selection of bio-inks to be considered. A key advantage of coaxial printing is its ease of manufacture, creating very long conduits in a minimal amount time. Deficiencies are the difficulty in creating complex, bifurcated, anatomical structures, and the creation of multiple microscale layers seen in many native tubular organ structures.

Direct printing of cell-laden gels onto a rotating rod can serve as a method for creating hollow tubes, and this additive manufacturing technique can be considered as the inverse of traditional lathe machining practices. The rod provides a temporary support for the hydrogel and is removed once the structure is deemed to be self-supporting. The company Sichuan Revotek claim to have utilised this method in combination with an adipose stem cell-laden hydrogel to create artery mimics that were subsequently transplanted into monkeys, although no data has yet been published (Fig. 3f). A variant of rod printing combines it with the previously described coaxial printing. Hollow microtubes are printed onto a rotating glass rod to create a multifluidic tubular organ with a micro- and macrochannel [62] (Fig. 3g).

## Bio-inks for 3D printing tubular organs

As previously indicated the selection of a suitable bio-ink is critical to any 3D printing process. A bio-ink is a formulation of material(s) and biological molecules or cells processed using bioprinting technologies [63]. Typically, these are combinations of hydrogels and living cells that are implemented into 3D bioprinting hardware capable of creating cell-laden structures of pre-defined geometries [15, 34]. A key challenge for researchers aiming to 3D print tubular, and other organ, structures is the formulation of bio-inks whose properties, in their final embodiment, align as closely possible with the tissue type being fabricated. The key structural component of a bio-ink is the hydrogel, a polymer-based structures of hydrophilic nature, with the ability to swell in high water content surroundings. Hydrogels therefore provide the three-dimensional environment required for cells to adhere and grow [64].

Table 1 presents a summary of the ideal requirements of engineered tissues for one tubular organ type, vascular grafts and has been produced by the adaption of a table presented by Catto et al. [65] for the requirements of an ideal tissue engineered.

**Table 1:** Ideal requirements of engineered tubular tissues adapted from Catto et al. [65]

Biocompatibility	Non-toxic
	Non-immunogenic
	Not susceptible to infection
	Growth potential for paediatric patients
	Non-cytotoxic degradation products
Mechanical properties	Mechanical properties similar to native vessel to allow structural stability
	Adequate suture retention/neighbouring vessel integration
Processability	Low manufacturing costs
	Readily available with many different sizes
	Sterilisable
	Easy storage

A further consideration is that the hydrogel may be required to degrade at an equal rate to extracellular matrix secretion by cells, ensuring the structure can be maintained during host integration [61, 66]. In addition, hydrogel degradation products must not have a considerably deleterious effect on fabricated organs or the surrounding in vivo environment, in order to avoid immunological issues. A summary of the requirements of hydrogels for 3D bioprinting using bio-inks can be seen in Table 2.

**Table 2:** Hydrogel requirements for 3D bioprinting using bio-inks

3D Structure	High porosity, integrin-activated, stiffness
Viscosity	Shear stress: shear-thinning/shear-thickening
Surface tension	Retention inside nozzle until printing. Limited spreading, spraying, spilling upon printing
Gelation	Rapid gelation via cross-linking, shape retention
Physical properties	Molecular mass, concentration, composition
Cell integration	Minimal viability loss during printing due to nozzle shear stress. Cell differentiation, proliferation, growth, tissue formation

Hydrogels can be broadly classified into two categories: natural and synthetic. Generally, natural hydrogel materials offer high biocompatibility and biodegradability whilst innately having the structure to support cell migration, adhesion, maintenance and growth with the major drawback of lacking the mechanical strength to support their own weight. Conversely, synthetic materials possess the mechanical strength to retain shape and structure but generally suffer from poor biocompatibility and are non-biodegradable.

### Natural materials

Of the many natural materials used within hydrogels and bio-inks, alginate is one of the predominant used in bioprinting. Alginate is a natural polysaccharide derived from the cell wall of brown algae, a linear copolymer consisting of guluronic acid and mannuronic acid, in differing ratios depending on batch composition [67]. Alginate is favoured in bioprinting environments due to its ability to form biocompatible, biodegradable and printable hydrogel at room temperature in addition to its low cost. However, alginate is known to be bio-inert and suffers from low cellular adhesion and slow degradation kinetics, resulting in unfavourable cellular proliferation and differentiation [68].

Alginate is particularly suited to extrusion-based bioprinting due to the ease of cross-linking and the wide range of possible concentrations giving rise to mechanically stable structures. Alginate can be extruded in either precursor or pre-crosslinked form, where alginate is mixed with a low concentration of cross-linker to improve printability [69]. Printed alginate can be strengthened further by the addition of cross-linker (typically 100–200 mM CaCl$_{2}$). These properties have led to the use of alginate-based bio-inks by different research groups for the creation of cell-encapsulated printable tubular structures. Tan and Yeong vertically printed tubular constructs [49], and Gao et al. and Gao et al. extruded alginate to fabricate the previously described coaxially printed vascular structures [62, 70].

Collagen too can be used as the hydrogel component of a bio-ink. Collagen is a protein that exists in a triple-helix arrangement of polypeptides [71] and is the most abundant structural protein in the human body, primarily located in the extracellular matrix (ECM) of connective tissues. Collagen exists in many types, distinguished by the three-dimensional structures that are formed. It should be noted that the type of ECM collagen can vary even within the same tissue, with collagen I, III and IV present in the vasculature [72].

The fibrillar Type I collagen is the most commonly used type in 3D bioprinting. Collagen has the ability to allow cell adhesion and enhance cell attachment and proliferation due to the presence of RGD (asparagine–glycine–aspartic acid) residues, allowing integrin binding [13]. Collagen is a biodegradable protein with low toxicity and minimal cross-species immunological reactions. All of these factors promote collagen as a suitable material to be used in 3D bioprinted constructs as a scaffold material [73].

As collagen is of low mechanical strength, it is mainly integrated into the bioprinting of tubular structures as a medium for cell encapsulation, similar to alginate, but with the aforementioned enhanced biocompatibility. A key advantage of collagen is that the bio-ink can be tailored contain the ECM components and cells and that are present in the tissue being mimicked, an approach used in casting methods of Weinberg and Bell [30].

Other natural materials that are commonly used in bioprinting include agarose, fibrin, gelatin, and hyaluronic acid, amongst others.

### Synthetic materials

Of all synthetic hydrogel constituents, pluronic F-127 is amongst the most commonly used, largely in part to its approved use in humans by the FDA [74]. Chemically, pluronics are triblock copolymers consisting of two hydrophilic polyethylene glycol (PEG) blocks at either end of a hydrophobic polypropylene glycol (PPG) block. Pluronic is a trademark name for poloxamers, of which there are many types, named by a letter (L for liquid, P for paste, F for flake/solid) followed by a two- or three-digit numerical value, representative of chain length and molecular weight.

Pluronic F-127 forms micelles in solution once a certain concentration is reached, known as the critical micelle concentration (CMC). Upon reaching the CMC, the solution transitions to a gel phase, however, this gel is of low mechanical strength. The concentration of pluronic F-127 can be controlled in such a way as to manipulate the gelation temperature and hence printability during extrusion-based bioprinting. A minimal concentration of 15 wt% [75] is required for gelation of pluronic F-127; typically a concentration of 25–40% w/v is utilised. This allows the storage of pluronic F-127 as a liquid at sub-room temperature, gel printing at room temperature and storage of printed scaffolds up to incubation temperature. Pluronic scaffolds can be thermo-degraded back to liquid state and washed away simply by lowering the temperature below the lower critical gelation temperature (LCGT) [76].

In addition, pluronics have been used in cell-printing operations, partially due to their low toxicity [77], and it has been shown that they can be printed with no excessively detrimental shear stress effects on encapsulated cells [78]. The aforementioned factors all indicate that pluronic F-127 is a suitable material for 3D bioprinting. However, it should be noted that the synthetic nature of pluronics naturally deems them non-bioactive, therefore deeming them unusable in environments where long-term cell viability is required [79]. This is in addition to the tendency for pluronic to dissolve in aqueous environments, leading to incompatibility with long-term cell culture conditions where scaffold structural support is essential. Gao et al implemented pluronic F-127 (40% w/v) in a core-shell coaxial printing configuration, with the aim of creating biological blood vessels (BBV). Recently, the Suntornnond group combined pluronic F-127 with gelatin methacrylate (GelMA) to create a biocompatible hydrogel with the ability to maintain shape integrity and capable of producing perfusable complex vascular-like structures upon printing. Printed hollow quadfurcated tubular structures supported human umbilical vein endothelial cell (HUVEC) proliferation and differentiation [80].

DNA-based hydrogel is also a novel and outstanding bio-ink for 3D printing. Compared to other synthetic materials, DNA-based hydrogels have many excellent characteristics, such as responsiveness, biocompatibility, shear-thinning and fast self-healing properties. Liu and Shu et al. achieved in situ multilayer three-dimensional living cell bioprinting using DNA-polypeptide hybrid supramolecular hydrogel. In this method, two bio-inks were co-printed through the dual-nozzle printer. One is the DNA-polypeptide solution mixed with living cells, and the other is the complementary DNA solution. It is noteworthy that the printed structures are intact and uniform without gaps between two layers, which is attributed to the self-healing property of the hydrogels. Moreover, the cells in the printed hydrogels have high viability reaching 98.8% [81]. Later, Liu et al. also developed a new “brick-to-wall” strategy to construct tissue-like structure based on pure DNA hydrogels. Compared to traditional supramolecular hydrogels, this pure DNA hydrogel is fully composed of stiff DNA duplexes and there is no chain entanglement in the network, resulting in the absence of the pores smaller than a certain size. It therefore has good permeability allowing growth factors and proteins to diffuse into the DNA hydrogels’ internal network. Exploiting this advantage, the group encapsulated different cells into separate hydrogel bricks and observed cell migration between them [82]. Although supermolecular DNA-based hydrogels [83–85] have yet to be explored in the context of tubular organ manufacture, their mechanical strength and capacity to form multiple layers, observed in tubular tissue, makes them a promising material for the future.

## Future challenges and prospects

If biofabricated tubular organs are to usurp current synthetic, donor and autologous implants, they will be required to closely match native human tissue in terms of anatomical accuracy, environmental responsiveness, mechanical properties, autoimmune acceptance, long-term patency and ultimately functionality. It is accepted that in attempting to attain this objective researchers should attempt to mimic healthy tissue architecture on the macro-, micro- and potentially nanoscales [6, 19, 86]. This represents a considerable technological challenge, and the complex nature of native tissue means that even the most innovative current techniques show promise they are only able to offer approximations of the healthy tissue they are intended to replace. Although the advent of new additive manufacturing technologies has enabled researchers to progress considerably over the previous decade, there still remains a large gap in terms of functionality between those devices created in the laboratory and the stringent clinical demands of an implanted protheses. Furthermore, in common with other new medical technologies a clear regulatory structure offering a route to market has yet to emerge [87, 88]. A further consideration is that the clinical needs of multiple patients may require bespoke 3D fabricated tissue urgently at the same time, thus placing stringent timescales and speed requirements on any production process [9]. The integration of automation into the process of tubular organ fabrication has the potential to address these manufacturing requirements [33].

Whilst the challenges in transferring from the laboratory bench to clinical use remains high, we predict that 3D biofabrication will have a crucial role in the future therapeutic treatment of tubular tissue disease and malfunction. The inherent advantage of using 3D bioprinting to create tubular organs is the capability to create the complex anatomical features seen in many tubular organ structures throughout the body. This has been demonstrated in the intricate perfusable vascular networks created by vasculature embedding and FRESH printing [50, 52, 53, 55]. Progress has also been facilitated by the growing array of bio-inks available to researchers, with developments in DNA-based hydrogels showing potential for use in tubular organ construction [82]. However, the microscale arrangement of multiple cellular and ECM layers, present in many native tubular organs, is still to be achieved using these methods. Other techniques, such as rod support printing, have the potential to create tubes that are composed of multiple layers of cell-laden hydrogel variants. Various levels of this type of cell-ECM organisation have also been demonstrated using other tissue engineering techniques, beyond 3D printing, such as sheet rolling [31–33], dip coating [34, 35] and post assembly culture reorganisation [7, 25, 36, 37]. Although extremely promising, such methods are currently limited in terms of the anatomical complexity that is required in many tubular organ reconstruction scenarios. Therefore, future developmental advances in tubular tissue biofabrication may reside in combining the advantages of spatial control provided by 3D printing with the cellular scale organisation control seen in other tissue engineering methods.
